# GMMA-based vaccine candidates against invasive nontyphoidal salmonellosis elicit bactericidal antibodies against a panel of epidemiologically relevant *Salmonellae*

**DOI:** 10.3389/fimmu.2025.1610067

**Published:** 2025-06-20

**Authors:** Daniele De Simone, Marika Pinto, Maria Grazia Aruta, Marta Benincasa, Martina Carducci, Roberta Di Benedetto, Francesco Citiulo, Miren Iturriza, Elli Mylona, Stephen Baker, Mariagrazia Pizza, Carlo Giannelli, Francesca Mancini, Rocío Canals, Omar Rossi

**Affiliations:** ^1^ GSK Vaccines Institute for Global Health (GVGH), Siena, Italy; ^2^ Cambridge Institute of Therapeutic Immunology and Infectious Disease, University of Cambridge, Cambridge, United Kingdom; ^3^ ASTAR Infectious Diseases Labs (ASTAR IDL), Singapore, Singapore; ^4^ Imperial College, London, United Kingdom

**Keywords:** GMMA, iNTS, *Salmonella*, SBA, vaccine, O-antigen, bactericidal

## Abstract

Systemic disease caused by nontyphoidal *Salmonella* (NTS) represents a major cause of death and morbidity, especially in young children in sub-Saharan Africa. No licensed vaccine is yet available, and an increase in antimicrobial resistance makes the development of a vaccine a global health priority. We are developing a bivalent formulation of *Salmonella* Typhimurium and *Salmonella* Enteritidis generalized modules for membrane antigens (GMMA)–based vaccine (iNTS-GMMA) and a trivalent formulation (iNTS-TCV) in which iNTS-GMMA is combined with the WHO-prequalified TYPHIBEV (Biological E, India) vaccine to prevent typhoid fever in addition to invasive NTS (iNTS) disease. Here, we measured the ability of antibodies induced by iNTS-GMMA and iNTS-TCV formulations in mice and rabbits to kill a broad panel of *Salmonella in vitro* in a complement-mediated fashion. These organisms include those causing invasive disease in Africa and Southeast Asia, global representatives causing gastroenteritis and other *S*. *enterica* serovars in addition to *S.* Typhimurium and *S.* Enteritidis. We characterized the O-antigen of the panel of isolates and demonstrated the sera functionality in both animal species against all isolates, providing evidence of the potential broad coverage of both GMMA-based formulations, which are currently undergoing testing in Phase I/II clinical trials.

## Introduction

*Salmonella* represents a major cause of morbidity and mortality, and organisms causing human disease are divided into typhoidal and nontyphoidal. Typhoidal *Salmonella*, *S.* Typhi and *S.* Paratyphi, causes typhoid and paratyphoid fever, respectively, and is collectively known as enteric fever. Non-typhoidal *Salmonella* (NTS) usually causes self-limiting gastroenteritis in high-income countries (1). However, among children under 5 years old in sub-Saharan Africa, NTS causes bloodstream infections, known as invasive NTS (iNTS) disease, associated with a high mortality rate (15%) (2). iNTS disease alone was estimated to have caused 535,000 illnesses and more than 77,000 deaths in 2017 (3). While there are licensed vaccines to prevent *S*. Typhi infections, no currently licensed vaccine can provide protection against NTS infections (4). Therefore, there is an urgent need for vaccines against iNTS disease, considering that these *Salmonellae* are usually associated with multidrug resistance (MDR) (5, 6). In addition, extensively drug-resistant (XDR) isolates make the treatment with antimicrobials a greater clinical challenge (7).

Immunity against *Salmonella* is serotype specific, and the O-antigen (OAg) portion of the lipopolysaccharide (LPS) represents a key target antigen for immunity. Several vaccine candidates against iNTS disease targeting the OAg are currently under development (8). Among these, a bivalent formulation of *Salmonella* Typhimurium and *Salmonella* Enteritidis generalized modules for membrane antigens (GMMA) as a delivery system of OAg has been proposed by GSK Vaccines Institute for Global Health (GVGH), known as iNTS-GMMA (9, 10). In addition, GVGH is developing a trivalent formulation (iNTS-TCV) in which iNTS-GMMA is combined with the WHO-prequalified Vi-CRM_197_ glycoconjugate (TCV) TYPHI-BEV^®^ by Biological E Ltd. (Hyderabad, India) to prevent typhoid fever (11) and iNTS. GMMA are outer membrane exosomes obtained from genetically modified organisms (12) to allow hyper blebbing and reduce the risk of systemic reactogenicity when injected into humans (13, 14). Vaccine-induced antibodies can trigger various effector functions (15), and, among them, the ability to activate complement-mediated killing could have a central role for *Salmonella*. Therefore, demonstrating *in vitro* the ability of antibodies to kill pathogens could represent an important indication of protective immunity (16); the gold standard for this evaluation is represented by a serum bactericidal assay (17).

The immunogenicity and the functionality of the antibodies raised in mice by *S.* Typhimurium and *S.* Enteritidis GMMA has been verified against vaccine-matched *S*. Typhimurium and *S*. Enteritidis strains, as well as their ability to protect in a mice challenge model (18). Both the iNTS-GMMA and iNTS-TCV vaccines are now being tested in clinical trials, with Phase I/II studies in healthy adults both in non-endemic and endemic populations (9, 10).

Among organisms causing iNTS disease, serovars Typhimurium and Enteritidis are the most prevalent (19). *S*. Typhimurium and *S*. Enteritidis belong to serogroups O:4 and O:9, respectively, accounting for 90% of NTS isolates from sterile sites (20). Moreover, other *Salmonella enterica* serovars share the same serogroup of *S*. Typhimurium and *S*. Enteritidis, like *S.* Derby (characterized by linkage α-Abe(1→3)Man to the common trisaccharide backbone of α-Man(1→4)-α-Rha-(1→3)-α-Gal-(1→2)) and *S.* Dublin (characterized by linkage α-Tyv(1→3)Man to the common trisaccharide backbone of α-Man(1→4)-α-Rha-(1→3)-α-Gal-(1→2)), respectively. Therefore, in the present study, we aimed to test sera raised both in mice and in rabbits with iNTS-GMMA and iNTS-TCV formulations to evaluate their ability to kill a panel of clinically relevant *Salmonella* originally isolated in different settings. We characterized the OAg of all the strains in terms of size, O-acetylation, and glucosylation, and then demonstrated the bactericidal activity of antibodies elicited by the candidate vaccines against the entire panel tested. These results suggest the potential of both vaccine candidates to broadly kill not only *Salmonella* Typhimurium and Enteritidis but also *S.* Derby and *S*. Dublin serotypes that share the same serogroup, respectively, independently of the small differences detected in the OAg.

## Materials and methods

### Bacterial strains selection and characterization

*Salmonella enterica* strains (**Table 1**) were obtained from the Novartis Master Culture Collection (*Salmonella enterica* serovar Typhimurium D23580 and *Salmonella enterica* serovar Enteritidis CMCC4314) (21) or purchased from ATCC (*Salmonella enterica* serovar Dublin and *Salmonella enterica* serovar Derby) or the National Collection of Type Cultures from the UK Health Security Agency (*S*. Enteritidis A1636, CP255, and D7795) or provided by the University of Cambridge collection (*S.* Typhimurium 1, 4, 12, 10433_3, 4/74, and A130), grown in Luria–Bertani (LB), and stored frozen at −80˚C in 20% glycerol stocks until use. An overday culture was started from a loop of material from the glycerol stock in 5 ml of LB medium and incubated at 37°C for 6h, stirring at 180 rpm. The overnight bacterial cultures were then used to start a 50 ml suspension in LB, which was afterwards incubated overnight (16–18 h) at 37°C with 180 rpm agitation in an orbital shaker. The OAg portion of the LPS was then directly extracted from all *Salmonella* isolates, normalized to the same final OD, and the total amount was quantified by high-performance anion-exchange chromatography coupled to a pulsed amperometric detector (HPAEC–PAD), as previously described (22). Briefly, the growth culture was subjected to 2% acetic acid hydrolysis (5h at 100°C), and the cell supernatant, containing free OAg, was collected after centrifugation. Lower molecular weight impurities were removed, and the cell supernatant was concentrated by ultrafiltration, using an Amicon-10 kDa device (Merck Millipore, St. Louis, Missouri, United States). Protein and nucleic acid impurities were coprecipitated in 20 mM citrate buffer. Nucleic acids were further removed by precipitation in 18 mM Na_2_HPO_4_, 24% ethanol, and 200 mM CaCl_2_. The OAg was recovered in water by a second ultrafiltration 10-kDa step, and finally a last ultrafiltration 30-kDa step was performed to remove the core from the OAg. All the OAg were characterized using the following analytical methods: size-exclusion high-pressure liquid chromatography (HPLC–SEC), HPAEC–PAD, and Hestrin assay to assess acetylation (23).

**Table 1:** *Salmonella s*trains used in this study and their abbreviations.

Species and serovars	Strain (sequence type)	Source	Reference(s)
*Salmonella enterica* serovar Typhimurium	D23580 (ST313)	Isolated from human blood in Malawi.	(27)
*Salmonella enterica* serovar Typhimurium	1,4,12 (ST34)	Isolated from human blood in Vietnam.	(28)
*Salmonella enterica* serovar Typhimurium	10433_3 (ST313)	Isolated from human blood in Democratic Republic of the Congo.	(29)
*Salmonella enterica* serovar Typhimurium	4/74 (ST19)	Isolated from isolated from the bowel of a calf.	(30)
*Salmonella enterica* serovar Typhimurium	A130 (ST313)	Isolated from human blood in Malawi.	(27)
*Salmonella enterica* serovar Enteritidis	CMCC4314, ATCC4931	Isolated from a case of gastroenteritis in Copenhagen.	(31)
*Salmonella enterica* serovar Enteritidis	A1636	Isolated from human blood in Malawi.	(32, 33)
*Salmonella enterica* serovar Enteritidis	CP255	Isolated from human blood in Democratic Republic of the Congo.	(33)
*Salmonella enterica* serovar Enteritidis	D7795	Isolated from human blood in Malawi	(32, 33)
*Salmonella enterica* serovar Derby	ATCC6960	Isolated from tank water and pork pies	(31, 34)
*Salmonella enterica* serovar Dublin	ATCC39184, SL1438	Derived from existing strain, S4454	(31, 34)

Panel includes global and invasive *S*. Typhimurium and *S*. Enteritidis isolates from Africa and Southeast Asia, and *S*. *enterica* serovars other than *S*. Typhimurium m and *S*. Enteritidis.

### Formulation tested and animal experiments

GMMA was purified from *S*. Typhimurium and *S*. Enteritidis Δ*tolR* Δ*msbB* Δ*pagP*–producing strains and fully characterized as previously described (24).

Groups of eight female CD1 mice (5–6 weeks old) were immunized intraperitoneally with *S*. Typhimurium GMMA (2.5 µg) or *S*. Enteritidis GMMA (2.5 µg OAg) in 500 µl of saline solution at a 4-week interval. Groups of 10 female CD1 mice (5–6 weeks old) were immunized twice intraperitoneally with 1.0 µg OAg/dose of *S*. Typhimurium GMMA plus 1.0 µg OAg dose of *S*. Enteritidis GMMA in case of iNTS-GMMA formulation and the same amount of *S*. Typhimurium/*S*. Enteritidis GMMA plus 1.25 µg of fVi polysaccharide in the case of iNTS-TCV, twice at a 4-week interval. Groups of eight female New Zealand white rabbits were immunized intramuscularly twice, 4 weeks apart, with 20 µg of *S*. Typhimurium OAg/dose, 20 µg of *S*. Enteritidis OAg/dose in case of iNTS-GMMA formulation and with the same amount of *S*. Typhimurium and *S*. Enteritidis GMMA plus 25 µg of fVi/dose polysaccharide in case of iNTS-TCV. All formulations contained Alhydrogel. Blood samples from all studies were collected from the immunized animals on day 42 (14 days after the second immunization). The routes of immunization and vaccine doses for the two animal species were selected based on dose-ranging studies to induce optimal immunogenicity (data not shown).

Animal experiments were carried out at Charles River Laboratories (France) and Toscana Life Sciences (Italy). All animal experiments were performed in accordance with relevant national and international legislation (Italian Legislative Decree 26/2014 and European Directive for the Use of Animals for Scientific Purposes 2010/63) and the GSK Policy on the Care, Welfare, and Treatment of Animals. All animal protocols were reviewed by the local Animal Welfare Body and approved by the Ministry of Health, according to the above-mentioned legislation. GSK is committed to the replacement, reduction and refinement of animal studies (3Rs). Non-animal models and alternative technologies are part of our strategy and are employed where possible. When animals are required, the application of robust study design principles and peer review minimizes animal use, reduces harm, and improves benefit in studies.

### Assessment of bactericidal activity by luminescence-based serum bactericidal assay

All strains listed in **Table 1** were grown overnight at 37°C in 5 ml of Luria–Bertani (LB) medium, stirring at 180 rpm. The overnight bacterial suspensions were then diluted in 7 ml of fresh LB to an optical density at 600 nm (OD600) of 0.05 and incubated at 37°C with 180 rpm agitation in an orbital shaker until they reached 0.20–0.25 OD600. Baby (3- to 4-week-old) rabbit complement (Cederlane—CL3441-S100—Euroclone, Canada, at a final concentration of 50% for assays performed on *Salmonella enterica* serovars Typhimurium and Enteritidis strains and 15% for assays performed on *S. enterica* serovars Dublin and Derby) was stored in frozen aliquots and thawed immediately prior to use. Phosphate-buffered saline (PBS) at pH 7 was used for serum and bacterial dilutions for all *S*. Typhimurium and *S*. Enteritidis strains, whereas LB was used as a buffer assay for *S.* Dublin and *S*. Derby.

Serum bactericidal assay based on luminescence was performed in a 96-well plate (Corning) as previously described (25). Briefly, sera collected at day 42 were heat-inactivated (HI) at 56°C for 30 min and serially diluted in PBS (or LB in the case of *S.* Dublin and *S.* Derby) directly in the SBA plate (25 µl/well). The starting dilution of each serum in the assay was then followed by threefold dilution steps up to 7 dilution points, plus one control well with no sera added, which represents the control for nonspecific complement killing. Log-phase cultures (OD600 = 0.20–0.25) were prepared as described above and diluted to approximately 1 × 10^6^ colony-forming units (CFUs)/ml in PBS. The luminescence at T0 was measured by diluting the appropriate volume of bacteria in four different replicates in PBS and mixing at 1:1 (v:v) with BacTiter-Glo Reagent (Promega, Southampton, UK) for 5 min at room temperature on an orbital shaker; the luminescent signal was detected by a luminometer (Synergy HT, Biotek, Swindon, UK). Seventy-five µl/well of reaction mix constituted by target bacterial cells (10 µl/well containing approximately 20–25000 bacteria), BRC (50 µl/well for all strains except *S*. Dublin and *S*. Derby, or 15 µl/well for *S*. Dublin and *S*. Derby), and PBS or LB medium (15 µl/well or 50 µl/well in the case of *S*. Dublin and *S*. Derby, respectively) were added to each well of the SBA plate containing HI serum dilutions (final reaction volume 100 µl), mixed, and incubated for 3h at 37°C. At the end of the incubation (T180), the SBA plate was centrifuged for 10 min at 4000 × g at room temperature. The supernatant containing ATP derived from dead bacteria and SBA reagents was discarded, and the remaining live bacterial pellets were resuspended in PBS (100 µl/well), transferred to a white round-bottom 96-well plate (Greiner), and mixed at 1:1 (v:v) with BacTiter-Glo Reagent (Promega, Southampton, UK). The reaction was incubated for 5 min at room temperature on an orbital shaker, and the luminescent signal was detected.

A four-parameter nonlinear regression was applied to raw luminescence (no normalization of data was applied) obtained for all the serum dilutions tested for each serum; an arbitrary serum dilution of 10^15^ was assigned to the well containing no sera. Fitting was performed by weighting the data for the inverse of luminescence^2^ and using GraphPad Prism ver. 9 software (GraphPad Software). Results of the assay are expressed as the IC50, represented by the reciprocal serum dilution that is able to reduce the luminescence signal by 50% compared to the negative control (and thus causes 50% growth inhibition). Titers lower than the minimum measurable assay were assigned a value of half of the first dilution of sera tested.

Statistical analysis was performed using GraphPad Prism ver. 9. Comparison between two groups was performed applying nonparametric t-test (Mann-Whitney test).

## Results

### Selection and phenotypic characterization of bacterial strains

The OAg represents a key target of the immune response induced by non-typhoidal *Salmonella* (NTS), and several vaccine candidates are under development to deliver the OAg O:4 from *Salmonella enterica* serovar Typhimurium (*S*. Typhimurium) and O:9 from *S. enterica* serovar Enteritidis (*S*. Enteritidis). Understanding the ability of the induced antibodies to kill a broad panel of organisms that share a similar OAg is crucial to understanding the potential to protect the vaccine candidates. We previously showed the ability of *S*. Typhimurium GMMA and *S*. Enteritidis GMMA to induce antibodies in mice able to kill *Salmonella* with homologous OAg (18). Here, we selected a panel of *Salmonella* that included *S.* Typhimurium and *S*. Enteritidis from Africa and Asia, which cause invasive NTS (iNTS) disease, as well as *S. enterica* serovars Derby and Dublin, which are not included in the vaccine candidates but share the same serogroups O:4 and O:9, respectively, and global representatives causing diarrheal disease. Because the OAg may elaborate some structural variation between *Salmonella* isolates despite being of the same serogroup, and this might impact the ability of the elicited antibodies to kill the bacteria by SBA, the OAg of all isolates was extracted and fully characterized in terms of saccharide size, O-acetylation, and glucosylation level (**Table 2**).

**Table 2:** Characterization of OAg extracted from different Salmonella strains: OAg density, size, O-acetylation, and glucosylation percentage.

*Salmonella strain*	*OAg µg/OD*	*Size*		OAc %*	Glc %*
		High MW kDa	Medium MW kDa		
*S*. Typhimurium D23580	686	–	21	129	18
*S*. Typhimurium 1,4,12	408	92	22	106	30
*S*. Typhimurium 10433_3	566	–	24	120	39
*S*. Typhimurium 4/74	476	96	25	118	62
*S*. Typhimurium A130	530	91	21	83	13
*S*. Enteritidis CMCC4314	699	–	23	<8	16
*S*. Enteritidis A1636	413	–	23	28	13
*S*. Enteritidis CP255	445	90	24	22	23
*S*. Enteritidis D7795	661	–	24	23	21
*S.* Derby NCTC 1720	37	–	25	<28	18
*S. Dublin SL1438*	*456*	*-*	*27*	*60*	*18*

*%OAc and %Glc are expressed as molar ratio with respect to the OAg repeating unit.

Overall, all *S.* Typhimurium and *S.* Enteritidis isolates expressed comparable OAg at the same OD, except for *S.* Derby that expressed a lower amount. All *Salmonella* strains tested contained an OAg population with an average MW of approximately 20 kDa (medium MW), while strains *S*. Typhimurium 1,4,12, *S*. Typhimurium 4/74, *S*. Typhimurium A130 and *S*. Enteritidis CP255 demonstrated a bimodal population by presenting an additional population with an average MW of ~90 kDa (high MW). All five *S.* Typhimurium expressed OAg with a higher percentage of O-acetylation (83% to 120%) compared with the four *S.* Enteritidis strains and *S.* Derby (22%–28%, and 8% in the case of *S.* Enteritidis CMCC4314); 60% O-acetylation was instead observed for *S*. Dublin. Last, most of the *Salmonella* expressed a percentage of glucosylation between 13% and 23%, except for *S*. Typhimurium 1, 4, 12, and *S*. Typhimurium 10433_3 with 30% and 39%, respectively, and for *S*. Typhimurium four of 74 with 62%.

### Assessment of the ability of sera to induce complement-mediated bactericidal activity

To evaluate the functional activity of antibodies elicited by iNTS-GMMA and iNTS-TCV candidate vaccines, sera from mice and rabbits obtained by immunizing with single NTS GMMA and both vaccine candidates were tested by luminescence-based serum bactericidal assay (L-SBA) against the panel of vaccine-homologous and vaccine-heterologous sero-epidemiologically relevant *Salmonella*.

**Figure 1:**
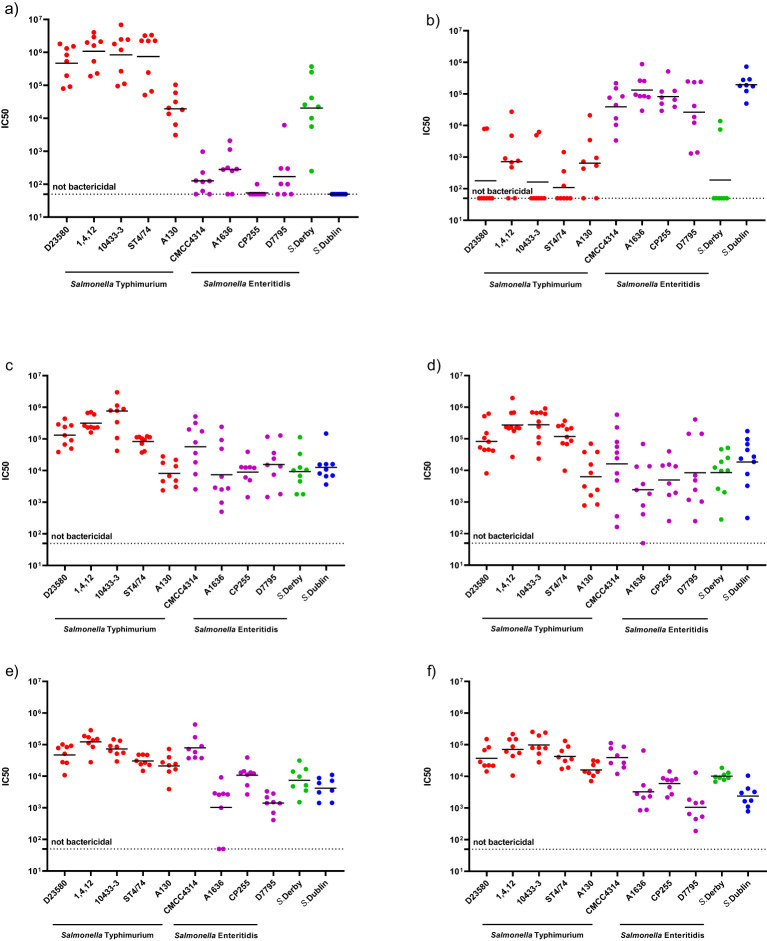
Serum bactericidal activity against a panel of *Salmonella* strains induced by different GMMA-based vaccine formulations: with mice sera elicited by monovalent *S*. Typhimurium GMMA/Alhydrogel **(A)**, with mice sera elicited by monovalent *S*. Enteritidis GMMA/Alhydrogel **(B)** with mice sera elicited by iNTS-GMMA **(C)**, with mice sera elicited by iNTS-TCV **(D)**, with rabbits sera elicited by iNTS-GMMA **(E)**, with rabbits sera elicited by iNTS-TCV **(F)**, respectively.

Sera raised in mice against mono-component NTS GMMA (*S*. Typhimurium or *S*. Enteritidis GMMA) were able to kill all strains sharing the same serogroup. Indeed, all the *S*. Typhimurium strains and *S.* Derby strains, which belongs to serogroup O:4, were killed by *S*. Typhimurium GMMA-induced antibodies, with no or very low SBA activity detected against *S*. Enteritidis strains and *S*. Dublin (**Figure 1A**). In contrast, sera elicited by monovalent *S*. Enteritidis GMMA demonstrated a high bactericidal titer against the four *S.* Enteritidis and *S.* Dublin, which belong to the same serogroup O:9, with no or minimal activity against *S*. Derby and the *S*. Typhimurium (**Figure 1B**). These results suggest the ability of the monovalent GMMA formulations to kill strains possessing the same serogroup, independently of the variations in OAg size and chemical features, such as the degree of either glycosylation or O-acetylation.

Next, we tested the functionality of the antibodies raised in mouse (**Figures 1C, D**) and rabbit models (**Figures 1E, F**) by iNTS-GMMA and iNTS-TCV vaccine formulations against the same panel of bacterial isolates. Both iNTS-GMMA and iNTS-TCV raised immune sera were able to kill all *S.* Typhimurium and *S*. Enteritidis panels as well as *S*. Derby and *S*. Dublin, suggesting the ability of both vaccine formulations, in two animal species, to induce antibodies able to kill a broad panel of vaccine homologous and vaccine heterologous strains. Comparing the IC50 induced by both multicomponent vaccine formulations against the same strain, no differences in terms of bactericidal activity were observed, thus suggesting no interference of the components (**Supplementary Figure S1**).

## Discussion

In this study, we assessed the ability of sera raised against a bivalent formulation of *S*. Typhimurium and *S*. Enteritidis GMMA-based vaccine (iNTS-GMMA) and a trivalent combination of iNTS-GMMA with the glycoconjugate Vi-CRM_197_ (iNTS-TCV) to kill, by L-SBA, a broad panel of *Salmonella* sharing the same serogroup. Prior to evaluating the functionality of antibodies, we characterized the OAg of all organisms in terms of saccharide size, O-acetylation, and degree of glucosylation level.

OAg characteristics are similar among isolates sharing the same serogroup. However, some differences are observed as investigated in this study. Since bactericidal activity was observed against all organisms sharing either O:4 or O:9 using sera raised against *S*. Typhimurium or *S*. Enteritidis GMMA, respectively, we conclude that the antibodies were able to kill organisms sharing the major OAg independent from the minor differences observed in characterization. In contrast, only a minimal bactericidal activity induced by sera elicited against *S*. Typhimurium-GMMA on *S*. Enteritidis and vice versa. This low bactericidal activity may be explained by minimal cross-reactivity of anti-OAg antibodies and/or by bactericidal activity mediated by anti-GMMA protein antibodies. Indeed, anti-GMMA protein antibodies have been previously shown to kill *Salmonella* (26), although this was at least two logs lower compared to the killing mediated by anti-OAg antibodies. While this represents a limitation of the current study, it should not affect the overall conclusions. Indeed, since GMMA was tested in all formulations, the anti-GMMA antibodies induced should similarly affect the bactericidal response for all strains.

A full dissection of the anti-GMMA protein response as well as the ability of the vaccine to induce cellular-mediated immunity will be addressed in future studies with human samples. When *S*. Typhimurium and *S*. Enteritidis GMMA are combined in a vaccine formulation, they elicit a humoral response able to kill *Salmonella* of the same serogroup, including vaccine homologous isolates and other vaccine heterologous isolates, both in mice (**Figures 1C, D**) and in rabbits (**Figures 1E, F**), suggesting the potential of iNTS-GMMA and iNTS-TCV as candidate vaccines to protect against a broad range of *Salmonella*. Moreover, even when GMMA is combined with Vi glycoconjugate, no differences in terms of the magnitude of bactericidal activity were observed, suggesting no interference. A further limitation of this study is that we have not demonstrated that the *in-vitro* functional response translates into *in-vivo* protection against all the isolates tested. However, in a previous study (18), we demonstrated that both *S.* Typhimurium and *S.* Enteritidis GMMA were able to provide protection against isolates displaying O:4 and O:9; in the same study, a good correlation between SBA results and protection was observed, suggesting that the functional response observed *in vitro* in the current study might translate into *in-vivo* protection against challenge with the various isolates. Although results are expressed in SBA titers and thus serum dilution, a full quantitative comparison of titers against different organisms was not possible due to the different sensitivity to killing, despite the percentage of heterologous complement used in the assay being the same for all *S*. Typhimurium and *S*. Enteritidis. Similarly, a further small limitation of the study is represented by the lack of a full comparison between the responses induced by sera raised against vaccine candidates and the ones that would have been induced by GMMA generated from matched heterologous strains. Translatability of results obtained in animals to humans’ debate, especially against pathogens for which a correlate of protection has not been established; however, since similar results are observed when testing sera raised in mice and rabbits, which possess a different ability to engage TLR-activating components present in GMMA, results are promising.

In conclusion, both iNTS-GMMA and iNTS-TCV can mediate complement-mediated killing not only against vaccine-matched *Salmonella* but also against a broad panel of epidemiologically relevant heterologous *Salmonella*, including isolates associated with bloodstream infections and diarrheal disease, African and Southeast Asian representatives, and strains belonging to different *S*. enterica serovars. Such results, if confirmed in humans, should give confidence of a broad coverage of the candidate vaccines and support reducing the burden in iNTS disease.

## Data Availability

The original contributions presented in the study are included in the article/**Supplementary Material**. Further inquiries can be directed to the corresponding author.
