# Supplementary material for: GMMA-based vaccine candidates against invasive nontyphoidal salmonellosis elicit bactericidal antibodies against a panel of epidemiologically relevant Salmonellae
Source: Front Immunol. 2025 Jun 20;16:1610067. doi: 10.3389/fimmu.2025.1610067 (PMC12231430; doi:10.3389/fimmu.2025.1610067)
Supplement: Supplementary file 1 [file DataSheet1.docx]

GMMA-based vaccine candidates against invasive nontyphoidal salmonellosis elicit bactericidal antibodies against a panel of epidemiologically relevant *Salmonellae*

Daniele De Simone^1^, Marika Pinto^1^, Maria Grazia Aruta^1^, Marta Benincasa^1^, Martina Carducci^1^, Roberta Di Benedetto^1^, Francesco Citiulo^1^, Miren Iturriza^1^, Elli Mylona^3^, Stephen Baker^4^, Mariagrazia Pizza^2^, Carlo Giannelli^1^, Francesca Mancini^1^, Rocío Canals^1^, Omar Rossi^1*^

^1^GSK Vaccines Institute for Global Health (GVGH), Via Fiorentina 1, 53100 Siena, Italy

^2^Imperial College, Exhibition Road SW7 2AZ, London, UK

^3^ Cambridge Institute of Therapeutic Immunology and Infectious Disease, University of Cambridge, Cambridge, United Kingdom

^4^ A*STAR Infectious Diseases Labs (A*STAR IDL) Singapore

*** Correspondence:**
Dr Omar Rossi, [omar.x.rossi@gsk.com](mailto:omar.x.rossi@gsk.com)

Keywords: GMMA; iNTS; *Salmonella*; SBA; Vaccine; O-Antigen; bactericidal

**Figure 1S** SBA titers of sera elicited in mice (a-k) and rabbits (l-v) with iNTS-GMMA and iNTS-TCV against a panel of *S*. Typhimurium, *S*. Enteritidis or *S*. Derby and *S*. Dublin strains. Mann-Whitney test has been used in order to compare titers induced by the two formulations against the different strains.
